# Effects of a High-Protein/Moderate-Carbohydrate Diet on Appetite, Gut Peptides, and Endocannabinoids—A Preview Study

**DOI:** 10.3390/nu11102269

**Published:** 2019-09-21

**Authors:** Lea Tischmann, Mathijs Drummen, Blandine Gatta-Cherifi, Anne Raben, Mikael Fogelholm, Bolette Hartmann, Jens J. Holst, Isabelle Matias, Daniela Cota, Ronald P. Mensink, Peter J. Joris, Margriet S. Westerterp-Plantenga, Tanja C. Adam

**Affiliations:** 1 Department of Nutrition and Movement Sciences, Maastricht University Medical Centre, 6200 MD Maastricht, The Netherlands; 2 NUTRIM School of Nutrition and Translational Research in Metabolism, Maastricht University, 6200 MD Maastricht, The Netherlands; 3 Department of Endocrinology, University Hospital of Bordeaux, F-33607 Pessac, France; 4 INSERM, Neurocentre Magendie, Physiopathologie de la Plasticité Neuronale, U1215, F-33000 Bordeaux, France; 5 University of Bordeaux, Neurocentre Magendie, Physiopathologie de la Plasticité Neuronale, U1215, F-33000 Bordeaux, France; 6 Department of Nutrition, Exercise and Sports, University of Copenhagen, DK1017 Copenhagen, Denmark; 7 Department of Food and Environmental Sciences, University of Helsinki, FI-00014 Helsinki, Finland; 8 NNF Center of Basic Metabolic Research and Department of Biomedical Sciences, University of Copenhagen, 2200 Copenhagen, Denmark

**Keywords:** Protein, endocannabinoids, hunger, satiety, gut peptides, weight maintenance, obesity

## Abstract

Favorable effects of a high-protein/moderate-carbohydrate (HP/MCHO) diet after weight loss on body weight management have been shown. To extend these findings, associations between perception of hunger and satiety with endocannabinoids, and with glucagon-like peptide-1 (GLP-1) and polypeptide YY (PYY) were assessed. At approximately 34 months after weight loss, 22 female and 16 male participants (mean age 64.5 ± 5.9 years; body mass index (BMI) 28.9 ± 3.9 kg/m^2^) completed a 48 h respiration chamber study. Participants were fed in energy balance with a HP/MCHO diet with 25%:45%:30% or a moderate-protein/high-carbohydrate (MP/HCHO) diet with 15%:55%:30% of energy from protein:carbohydrate:fat. Endocannabinoids and related compounds, relevant postprandial hormones (GLP-1, PYY), hunger, satiety, and ad libitum food intake were assessed. HP/MCHO versus MP/HCHO reduced hunger perception. The lower decremental area under the curve (dAUC) for hunger in the HP/MCHO diet (−56.6% compared to MP, *p* < 0.05) was associated with the higher AUC for 2-arachidonoylglycerol (2-AG) concentrations (*p* < 0.05). Hunger was inversely associated with PYY in the HP/MCHO group (r = −0.7, *p* < 0.01). Ad libitum food intake, homeostatic model assessment for insulin resistance (HOMA-IR) and incremental AUCs for gut peptides were not different between conditions. HP/MCHO versus MP/HCHO diet-induced reduction in hunger was present after 34 months weight maintenance in the post-obese state. HP/MCHO diet-induced decrease of hunger is suggested to interact with increased 2-AG and PYY concentrations.

## 1. Introduction

In 2016, 39% of the adult population worldwide was considered overweight and 13% obese [1]. A positive energy balance is one of the most critical underpinnings for this development, posing a major risk for the development of chronic diseases including type-II diabetes (T2D) and cardiovascular disease [2].

While successful dieting and weight maintenance are essential to long-term improvements of metabolic disease, weight maintenance remains especially challenging. For weight reduction as well as long-term weight maintenance, dietary protein was suggested as being potentially helpful due to the notion that it appears to be more satiating than carbohydrates or fat in an acute setting [3,4,5,6,7], and therefore may support the reduction of food intake [8]. Additionally, a ketogenic state, induced by a short- or medium-term high-protein, low-carbohydrate condition [9,10], has been suggested as a contributor to protein-related appetite regulation. In addition to increased satiety, protein was shown to have sparing effects on fat-free mass (FFM) during weight reduction and weight maintenance [11,12], while energy expenditure increased [5,11,13,14].

The endocannabinoid system is critically involved in the regulation of energy balance and in the pathophysiology of metabolic disorders [15,16,17]. The system comprises endogenous lipids, the cannabinoid receptor 1 and 2 (CB1 and CB2) and the enzymatic machinery involved in the synthesis and degradation of endocannabinoids [15]. The best characterized endocannabinoids are the N-ethanolamide of arachidonic acids, known as anandamide (AEA) and the glycerol ester of arachidonic acid or 2-AG. The lipid derivatives and endocannabinoid-related compounds oleoylethanolamide (OEA) and palmitoylethanolamide (PEA) are synthetized together with AEA and share structural similarities, but do not bind to cannabinoid receptors. AEA and 2-AG both correlate positively with markers of obesity and metabolic disorders in humans [18,19,20]. OEA decreases appetite and favors weight loss and lipolysis by acting through peroxisome proliferator-activated receptor-α (PPAR-α), contrasting the metabolic effects of the endocannabinoid-dependent CB1 receptor activation [21], which has been linked to obesity promotion [17]. Pregnenolone (PREG), a neurosteroid precursor, has been suggested to be a negative regulator of endocannabinoids by experimental data [22] and thereby prevents overstimulation of the cannabinoid receptor [22]. While endocannabinoids have been assessed with regard to energy balance [17,23] and fat intake [20], the role of dietary protein in endocannabinoid signaling remains unknown.

In addition, satiety-related gut peptides such as GLP-1 and PYY, have been reported to be increased [24,25] in response to high-protein intake compared to high-carbohydrate or high-fat intake. Both GLP-1 and PYY have incidentally been linked to increased satiety and reduced food intake [26]. However, based on the literature, individual perception of appetite and appetite-related gut peptides was not consistently associated [27]. Therefore, the use of gut peptides as a direct biomarker for perceived appetite alone appears insufficient [28].

The present study aimed to assess the effects of a high-protein/moderate-carbohydrate (HP/MCHO) versus moderate-protein/high-carbohydrate (MP/HCHO) diet in the post-obese state after weight loss, on the association between perception and physiology of hunger and satiety in energy balance, in a controlled respiration chamber setting. Especially, the protein-content-related differences in concentrations of endocannabinoids and related compounds as well as their potential association with hunger and satiety perception were assessed. We hypothesized that also in the post-obese phase, a high-protein/moderate-carbohydrate diet would be more satiating compared to a moderate-protein/high-carbohydrate diet, evidenced by higher satiety ratings and lower hunger ratings, possibly associated with changes in concentrations of endocannabinoids and related compounds, and with increased satiety hormone concentrations.

## 2. Materials and Methods

The study was registered at ClinicalTrials.gov (NCT01777893), was performed in line with the Declaration of Helsinki, and was approved by the Medical Research Ethics Committee of Maastricht University Medical Centre (METC). All participants provided written informed consent for participation. The study was performed at Maastricht University from February 2017 until February 2018.

### 2.1. Participants

A subgroup of 40 participants was recruited from the PREVention of diabetes through lifestyle intervention and population studies in Europe and around the World (PREVIEW) cohort [29] at Maastricht University in the Netherlands, of which 2 dropped out due to a lack of time (Supplementary Figure S1). Sample size calculation was based on energy expenditure [30]. The PREVIEW intervention study (Prevention of Diabetes through lifestyle intervention and population studies in Europe and around the World, EU seventh Framework Program, grant agreement no. 31205) is a multinational, multicenter, 2 x 2 factorial, randomized controlled trial aimed at finding the most effective lifestyle intervention to prevent the development of T2D in predisposed individuals. Individuals were between 25 and 70 years of age and had a Body Mass Index (BMI) above 25 kg/m^2^. Pre-diabetes, which was defined by a fasting plasma glucose concentration between 5.6 and 6.9 mmol/L and/or a 2 h plasma glucose between 7.8 and 11.0 mmol/L following an oral glucose tolerance test (OGTT) [29], was an inclusion criterion for participation. Exclusion criteria for this respiration chamber sub-study were claustrophobia, smoking, and previous cardiovascular events, next to the exclusion criteria for the PREVIEW study [29]. Written informed consent was obtained from all participants before starting the experiment.

### 2.2. Experimental Design

Detailed information on the PREVIEW intervention study design, interventions, subject recruitment, primary and secondary endpoints, and baseline characteristics have been published before [29]. In short, an 8 week weight-reduction period by means of a low-energy diet was followed by 34 months of a randomized intervention comprising four treatment groups: MP/HCHO with moderate-glycemic index (GI), or a HP/MCHO diet with low-GI, combined with either moderate- or high-intensity physical activity in a parallel design. In close proximity to the last clinical investigation day (after 34 months) of the PREVIEW intervention, a subgroup of participants underwent a 48 h experiment in the respiration chamber to assess specific HP/MCHO intake-related aspects of hunger and satiety regulation. Participants arrived at the Metabolic Research Unit Maastricht (MRUM) research facilities in the morning after an overnight fast from 22:00 h the night before. The respiration chamber experiment started at 9:30 h. Participants had fixed bedtimes in the respiration chambers from 11:30 h until 7:30 h and were not allowed to sleep during the daytime or to exercise.

### 2.3. Anthropometric Measurements

Body weight and body composition (BOD POD^®^, Life Measurement Inc., Concord, CA, USA) were measured before the respiration chamber experiment was started. Height was measured using a wall-mounted stadiometer during screening.

### 2.4. Respiration Chamber

The respiration chambers are 14 m^3^ airtight rooms with a controlled climate and furnished with a bed, chair, table, intercom, TV, computer, sink, and toilet. Continuous fresh air ventilation at a rate of 70–80 L/min was used and measured with a dry gas meter (G6, gasmeterfabriek Schlumberger, Dordrecht, the Netherlands). O_2_ and CO_2_ concentrations were continuously measured by open-circuit ventilated indirect calorimetry, using dual pairs of infrared CO_2_ analyzer (ABB/Hartman and Braun Uras, Frankfurt a.M., Germany) and paramagnetic O_2_ analyzers (Servomex 4100, Crowborough, England and ABB/Hartman and Braun Magnos, Frankfurt a.M., Germany) [31]. Total energy expenditure (EE) was calculated according to the formula of Weir [32].

### 2.5. Diets and Energy Intake

Participants received either a MP/HCHO (15%:55%:30% of energy from protein:carbohydrate:fat) or a HP/MCHO (25%:45%:30% of energy from protein:carbohydrate:fat) diet, according to their previous intervention group during the PREVIEW study. To keep menus as comparable as possible, the basis of all meals was the same between groups, combined with either carbohydrate- or protein-rich food items. Participants were asked to pay special attention to their prescribed study diet during the week before the experiment. All meals were provided in energy balance according to individual energy requirements during the respiration chamber session. The daily energy requirement was estimated by calculating the basal metabolic rate (BMR) with the use of fat mass (FM) and fat-free mass (FFM), which was then multiplied by a physical activity index of 1.35, based on previous respiration chamber experiments [33]. Dietary intake was divided over three meals with 20% of the daily energy requirement for breakfast (at 9:15 h) and 40% for both lunch (at 13:00 h) and dinner (at 17:45 h). No other food products were allowed for consumption. Water consumption was allowed throughout the study at the participants’ convenience and unsweetened coffee or tea were served at several time points. For the ad libitum brunch after the 48 h in the respiration chamber, participants received a buffet style meal. This meal was the same for the two intervention groups and comparable to the chamber breakfast from the days before, but not diet-specific and including choices of carbohydrate- or protein-rich options. Total energy and macronutrient intake were evaluated afterwards.

### 2.6. Appetite Profile

Subjective perception of appetite comprising hunger, fullness, and satiety was measured by 100 mm anchored visual analogue scales (VAS) from “not at all” to “very” during day 2 in the respiration chamber [34]. Questionnaires were scored before and 30 min after each meal, as well as once in between the meals. In the case of a simultaneous blood draw, questionnaires were done before the blood draw. The incremental area under the curve (iAUC) was calculated for satiety and fullness perception, and the dAUC was calculated for hunger perception using the trapezoidal rule [35].

### 2.7. Metabolic Parameters

Fasting blood samples were taken from an antecubital vein by venipuncture on the first and last day of the respiration chamber study for the analysis of fasting glucose, insulin, β-hydroxybutyrate, and triacylglycerol. On the second day, a venflon catheter (Becton, Dickinson and Company, Franklin Lanes, NY, USA) was placed in the antecubital vein for fasted and postprandial blood collections throughout the day for collection of endocannabinoids and related compounds, glucose, insulin, GLP-1, and PYY. Blood samples were drawn directly before and 30, 60, 90, and 120 min after all three meals. Except for serum samples, all samples were immediately stored on ice, centrifuged for 10 min at 1500 g at 4 °C, immediately distributed in aliquots, and stored at −80 °C until analysis at the end of the study, enabling all samples from one participant to be analyzed in the same run. Serum samples were kept at room temperature for 30 min to allow clotting before centrifugation (10 min at 1500 g at 4 °C). For endocannabinoid and related compounds measurements, samples were collected immediately before, 60 min after meals, and 120 min after dinner. The AUC was calculated for the endocannabinoids and related substances. The iAUC was calculated for GLP-1, PYY, glucose, and insulin using the trapezoidal rule [35].

#### 2.7.1. Endocannabinoids and Endocannabinoid-Related Compounds

For endocannabinoid and endocannabinoid-related compound analysis, ethylenediaminetetraacetic acid (EDTA) tubes (Becton, Dickinson and Company, Franklin Lakes, NY, USA) were used. Syringes and EDTA tubes were ice-chilled before blood collection and storage cups were prepared with 1% phenylmethylsulfonyl fluoride (PMSF) solution (10 mg PMSF in 1 mL methanol) and 5% 1N hydrochloric acid at final concentration. Samples were snap frozen in liquid nitrogen immediately.

The extraction, purification, and quantification of AEA, PEA, OEA, and 2-AG from blood require a set of different biochemical steps as described previously [19,36]. Samples were then subjected to isotope-dilution liquid chromatography-chemical ionization-tandem mass spectrometric analysis. Mass spectral analyses were performed on a TSQ Quantum Access triple quadrupole instrument (Thermo-Finnigan, San Jose, CA, USA) equipped with an APCI source (atmospheric pressure chemical ionization) and operating in positive ion mode [37]. Pregnenolone was extracted from plasma by a simple solid-phase extraction method using reverse-phase C18 columns according to the method described in Vallée et al. [22] and analyzed with a GC-MS/MS (gas chromatography-tandem mass spectrometer) XLS Ultra Thermo mass spectrometer (Thermo-Finnigan, San Jose, CA, USA) via an AS3000 II autosampler.

#### 2.7.2. GLP-1 and PYY

For analyses of GLP-1 and PYY concentrations, EDTA-aprotinin tubes (Becton, Dickinson and Company, Franklin Lakes, NY, USA) with added dipeptidyl peptidase IV inhibitor (10 mL/L blood) were used. Syringes and EDTA-aprotinin tubes were ice-chilled before blood collection and samples were snap frozen in liquid nitrogen immediately. Total GLP-1 [38] and PYY3-36 concentrations were both determined using a radioimmunoassay method. For the PYY assay, a 125-Iodine label was used and data were analyzed with RIACALC (Pharmacia, Freiburg, Germany).

#### 2.7.3. Glucose and Insulin

Plasma for colorimetric glucose analysis (Roche Diagnostic Systems, Woerden, the Netherlands) was collected in sodium fluoride tubes (Becton, Dickinson and Company, Franklin Lakes, NY, USA). Serum for insulin analysis was collected in serum separator tubes (Becton, Dickinson and Company, Franklin Lakes, NY, USA). Samples were used to analyze fasting and postprandial insulin concentrations with a human insulin-specific radioimmunoassay (Linco Research, St Charles, MO, USA). Insulin sensitivity was estimated by calculating the HOMA-IR [39].

#### 2.7.4. β-Hydroxybutyrate

EDTA vacutainer tubes (Becton, Dickinson and Company, Franklin Lakes, NY, USA) were used to collect plasma samples for β-hydroxybutyrate concentrations analysis with gas chromatography-mass spectrometry (GC-MS) quantification (Varian factor four VF-5 ms, 15 mx 0.25 mm * 0.1 µM with GCMS ms 7890A-7000c, Agilent, Santa Clara, CA, USA).

#### 2.7.5. Triacylglycerol

Serum samples (Becton, Dickinson and Company, Franklin Lakes, NY, USA) were used to analyze fasting triacylglycerol (GPO Trinder; Sigma-Aldrich Corp., St. Louis, MO, USA).

### 2.8. Statistical Analysis

All statistical tests were performed using SPSS for Macintosh (Version 25; SPSS Inc., Chicago, IL, USA). Data are presented as means ± standard deviations (SDs), unless otherwise indicated. Significance was defined as *p* < 0.05. Differences between both groups before and during their respiration chamber stay were calculated using ANOVA, and repeated measures ANOVA with BMI or body-fat percentage as a covariate, if appropriate. Not normally distributed data, as determined with the Shapiro–Wilk test, were log-transformed and tested on normality again. Partial correlation coefficients were calculated to assess associations between protein intake, appetite, gut peptides, and endocannabinoids, adjusted for BMI and fat percentage, if appropriate.

## 3. Results

### 3.1. Study Participants

The subject characteristics for the MP/HCHO and HP/MCHO group at baseline of the respiration chamber experiment are summarized in Table 1. Twenty participants started the respiration chamber experiment in the HP/MCHO condition and 18 participants in the MP/HCHO condition. The two groups were not different with regard to age, anthropometric variables, and fasting glucose and insulin concentrations prior to the respiration chamber experiment. Only triacylglycerol (TAG) was higher in the MP/HCHO group at baseline.

### 3.2. 24-Hour Hunger and Satiety Perception

**Figure 1:**
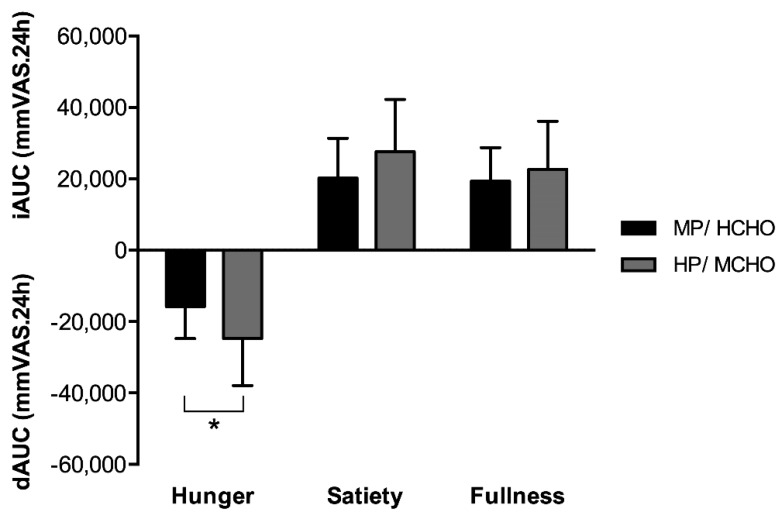
Visual analogue scales for appetite for MP/HCHO (black) and HP/MCHO (grey) groups. Hunger is presented as dAUC, fullness and satiety as iAUC. * *p* < 0.05. Values are means ± SD. Differences between groups were calculated with a One-Way ANOVA. MP/HCHO = moderate protein/high carbohydrate; HP/MCHO = high protein/moderate carbohydrate; iAUC = incremental area under the curve; dAUC = decremental area under the curve; mmVAS = millimeter visual analogue scale.

Baseline appetite profile was not different between conditions. Hunger perception (VAS) based on the decremental area under the curve during the whole day was significantly lower in the HP/MCHO diet compared to the MP/HCHO diet (−56.6%, F = 5.89, *p* < 0.05, Figure 1). Looking at the individual meals, specifically breakfast (−83%; F = 10.30, *p* < 0.01) seemed to have contributed to the overall difference between the intervention groups. The differences in hunger ratings could not be explained by differences in energy balance (MP/HCHO: 0.2 ± 0.9 megajoule (MJ), HP/MCHO: −0.5 ± 0.9 MJ; F = 6.03, *p* < 0.05) [30] between the two intervention groups. There were no differences in satiety or fullness ratings between the two groups (Figure 1).

### 3.3. Metabolic Parameters

#### 3.3.1. Endocannabinoids and Related Compounds Concentrations Throughout the Day

**Figure 2:**
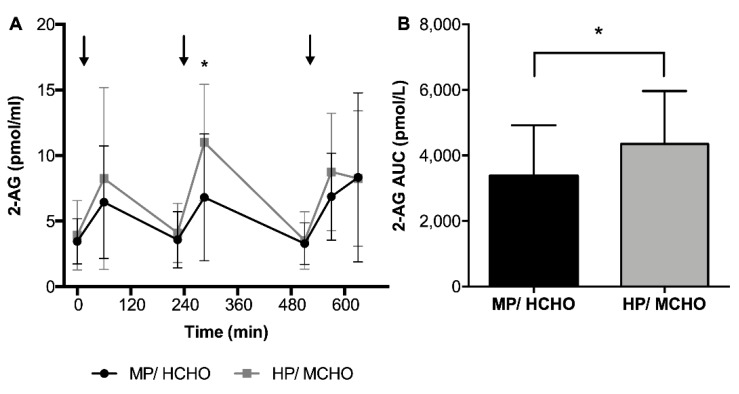
(**A**) Postprandial responses and (**B**) area under the curve (AUC) of 2-AG in the MP/HCHO (black) and HP/MCHO (grey) group. Arrows indicating timepoint of meals. Values are means ± SD. MP/HCHO = moderate protein/high carbohydrate; HP/MCHO = high protein/moderate carbohydrate; 2-AG: 2-arachidonoylglycerol. * *p* < 0.05.

**Figure 3:**
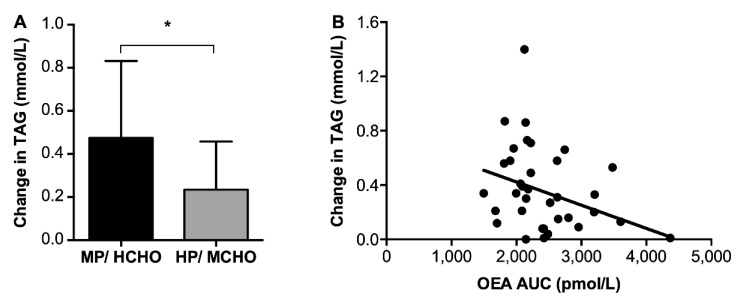
(**A**) AUC of change in TAG over 48 h in the MP/HCHO (black) and HP/MCHO (grey) group and (**B**) inverse association of OEA concentrations throughout the day and the change in TAG over 48h. Values are means ± SD. MP/HCHO = moderate protein/high carbohydrate; HP/MCHO = high protein/moderate carbohydrate; TAG: triacylglycerol; OEA: oleoylethanolamide. * *p* < 0.05.

2-AG showed increased concentrations 60 min after each meal, then decreasing back to baseline concentrations before the next meal (Figure 2). Around lunch, there was a significant time-by-treatment interaction for 2-AG (F = 6.61, *p* < 0.05). In addition, the AUC of 2-AG was significantly higher in HP/MCHO compared to MP/HCHO (4351 ± 1616 pmol/L versus 3368 ± 1552 pmol/L, F = 4.67, *p* < 0.05) and the post-meal change in 2-AG during dinner was positively related to the change in hunger (r = 0.37, *p* < 0.05). Post-meal changes of 2-AG were not related to changes in glucose, insulin, GLP-1, or PYY during any of the meals or to body weight or body composition. The postprandial change in 2-AG after dinner was positively associated with the change in pleasantness ratings (r = 0.425, *p* < 0.05). There was no clear meal associated pattern or higher protein/moderate carbohydrate intervention related effect in other endocannabinoids and related compounds. However, we observed that OEA concentrations (AUC) were inversely associated with the change in TAG (Figure 3) throughout the 48 h of the experiment (r = −0.40, *p* < 0.05).

#### 3.3.2. Gut peptides, Glucose, and Insulin Concentrations Throughout the Day

**Figure 4:**
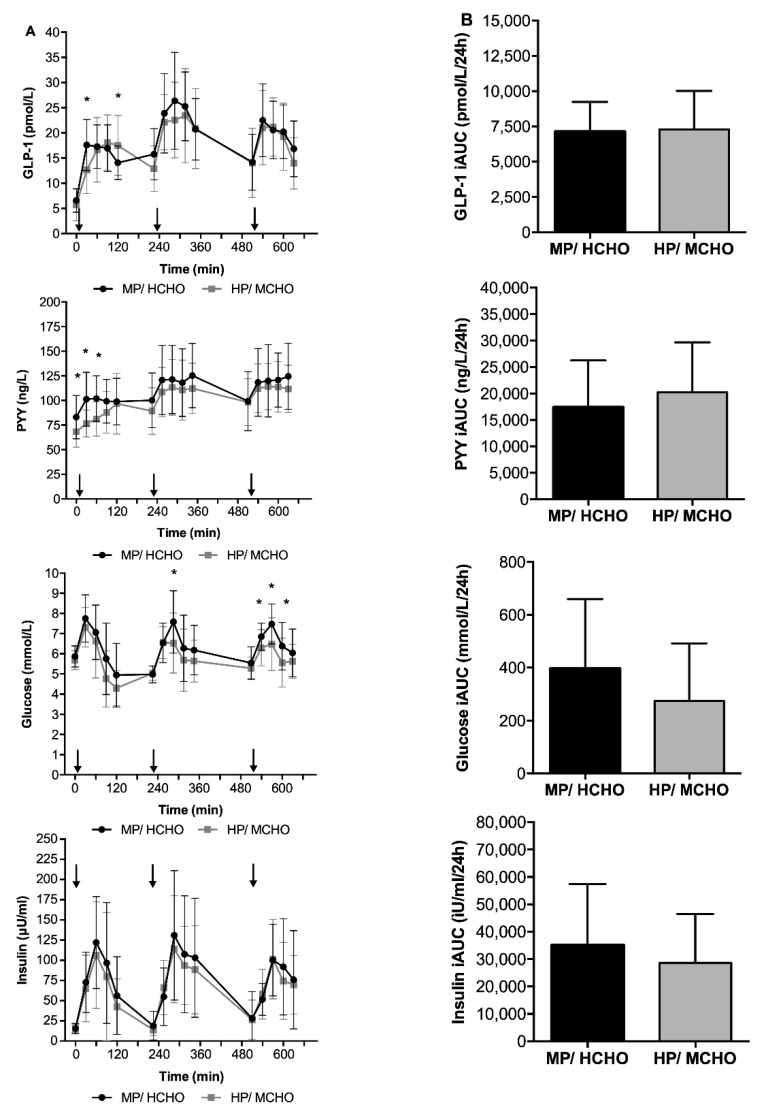
(**A**) Postprandial responses over time and (**B**) incremental area under the curves of GLP-1, PYY, glucose, and insulin in the MP/HCHO (black) and HP/MCHO (grey) group. Arrows indicating timepoint of meals. * *p* < 0.05. Values are means ± SD. Differences between groups were calculated with a One-Way ANOVA. MP/HCHO = moderate protein/high carbohydrate; HP/MCHO = high protein/moderate carbohydrate; GLP-1 = glucagon-like peptide 1; PYY = peptide YY; iAUC = incremental area under the curve.

**Figure 5:**
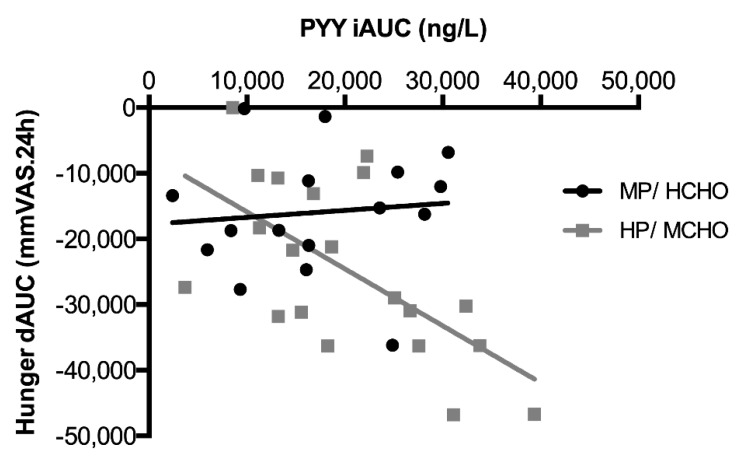
Inverse association of hunger and PYY concentrations throughout the day in the MP/HCHO (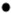) and HP/MCHO (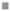) group. Regression lines are shown in black (MP/HCHO) and grey (HP/MCHO, r = 0.710, *p* = 0.001). MP/HCHO = moderate protein/high carbohydrate; HP/MCHO = high protein/moderate carbohydrate; iAUC = incremental area under the curve; dAUC = decremental area under the curve; PYY = peptide YY.

Postprandial plasma GLP-1 and PYY concentrations are shown in Figure 4. No significant differences in GLP-1 and PYY response, expressed as iAUC, were found between the HP/MCHO and the MP/HCHO group (Figure 4). In the complete group, the dAUC of hunger was inversely associated with the PYY iAUC (r = 0.40, *p* < 0.05) however this was especially the case in the HP/MCHO group. We found a significant interaction between group and PYY iAUC with regard to hunger ratings (F = 7.47, *p* < 0.05). In the HP/MCHO group, hunger (dAUC) was inversely associated with PYY (iAUC) concentrations (r = 0.71, *p* < 0.01; Figure 5), but not in the MP/HCHO group. GLP-1 was not associated with any of the subjective appetite assessments (hunger, fullness, and satiety). Neither PYY nor GLP-1 concentrations were associated with energy balance in any of the two intervention groups or with parameters related to glucose metabolism. Postprandial glucose concentrations were incidentally higher in the MP/HCHO group but both, glucose and insulin concentrations throughout the day (Figure 4) did not differ between the two intervention groups.

#### 3.3.3. Fasted β-Hydroxybutyrate and Triacylglycerol Concentrations

Plasma β-hydroxybutyrate concentrations, a marker for fat oxidation, were not different between dietary conditions (MP/HCHO: 90.05 ± 87.61 μg/mL and HP/MCHO: 117.57 ± 93.35 μg/mL). Baseline β-hydroxybutyrate was negatively associated with BMI (r = −0.35, *p* < 0.05) but was not associated with appetite perception or gut peptides. TAG concentrations increased in both intervention groups from pre- to post-respirations chamber measurement, but the increase was less pronounced (*p* < 0.05; Figure 3) in the HP/MCHO group compared to the MP/HCHO group (0.23 ± 0.22 mmol/L versus 0.47 ± 0.35 mmol/L, respectively).

### 3.4. Ad Libitum Energy Intake After Leaving the Chamber

Ad libitum energy intake (EI) (EI; MP/HCHO: 30.6% ± 10.4% energy requirement, HP/MCHO: 33.5% ± 12.4% energy requirement) as well as macronutrient content of meal choice (protein intake MP/HCHO: 18.5% ± 4.8% energy intake (%EI), HP/MCHO: 17.9% ± 4.0% EI; carbohydrate intake MP/HCHO: 50.2% ± 8.1% EI, HP/MCHO: 46.9% ± 7.5% EI; fat intake MP/HCHO: 30.3% ± 7.4% EI, HP/MCHO: 32.7% ± 6.0% EI) assessed during ad libitum brunch were not different between intervention groups. Ad libitum energy intake was not associated with excursions of gut peptides the day before or by insulin resistance, but it was negatively associated with total glucose concentrations (iAUC) of the previous day (r = −0.40, *p* < 0.05).

## 4. Discussion

This study demonstrates that a 48 hour high-protein/moderate-carbohydrate diet fed in energy balance under controlled conditions, reduced the perception of hunger and increased 2-AG concentrations after 34 months in a post-obese condition, compared to a moderate-protein, higher- carbohydrate diet. Hunger was inversely associated with PYY concentrations in the HP/MCHO group but not in the MP/HCHO group. High-protein/moderate-carbohydrate intake led to a blunted increase in TAG compared to moderate-protein/high-carbohydrate intake, while changes in TAG were inversely associated with OAE.

Reduced hunger and increased satiety or fullness have been previously found in studies applying acute high-protein diets, even in negative energy balance [13,14,25]. The decrease in hunger in the HP/MCHO versus the MP/HCHO group in the current study, however, did not translate into a decreased energy intake in the ad libitum brunch. Rather large differences in appetite ratings (e.g., ~40%) were necessary to actually result in differences in energy intake of e.g., ~20% in other studies, which applied a within subject design [8].

Among the endocannabinoids and related compounds studied, 2-AG showed a consistent meal-related pattern with highest concentrations one hour postprandially and lowest concentrations before the three meals. Interestingly, around all meals, the pattern of 2-AG concentrations was similar to that of concentrations in glucose, insulin, GLP-1, and PYY concentrations. However, in contrast to these parameters, 2-AG was the only one to be specifically affected by the higher-protein/moderate-carbohydrate content. Previous observations of plasma 2-AG dynamics around meals have been conflicting, from no meal-effect [36,40] to increased concentrations only after a hedonic meal, in both normal weight individuals [41] and in obese [42]. These data suggest that if food intake is driven by palatability of the food presented, rather than hunger, an increase in circulating 2-AG concentrations is observed, independent of the individuals’ BMI [41,42]. As higher 2-AG concentrations have been observed even before the actual consumption of palatable food, the authors have proposed an anticipatory role for this endocannabinoid in signaling the pleasure of the palatable food that is going to be eaten [41]. Regarding the possible hedonic component in endocannabinoid dynamics, desire to eat was not different between the intervention groups, and meals were described as equally pleasant in our study. However, postprandial changes in 2-AG were positively related to changes in pleasantness after dinner, therefore a hedonic effect cannot be excluded. No evidence can be found for a direct postprandial association of 2-AG with excursions of PYY and GLP-1 on an individual meal basis, suggesting a gut peptide-independent pathway of 2-AG in the current study. In contrast, 2-AG has been shown to be associated with GLP-1 [43] but not with appetite perception [41,43] previously. In the current study, however, postprandial changes of 2-AG were associated with changes in hunger after dinner. In our study, we characterized responses in participants who were still overweight, who had undergone a weight loss period for eight weeks followed by a 34 month period of weight maintenance before the current experiment was started. It is possible that the participants studied have a hedonic response different from lean, healthy-weight individuals, which in part may explain the postprandial increase in 2-AG concentrations observed in both intervention groups as 2-AG concentrations has been shown to be associated with BMI [18,19]. Finally, a likely origin of the changes in circulating 2-AG concentrations may be the intestine. Indeed, studies in rats have shown that gustatory stimulation with fat, without actual food intake, leads to an increase in both 2-AG and AEA content in the small intestine, which then further favor fat consumption through a positive feedback loop involving vagal afferents and the brainstem [44]. Accordingly, it has been shown that cheese with a specific fatty acid profile could influence plasma endocannabinoids in humans [45]. However, the role of other nutrients, especially protein on endocannabinoid concentrations, has never been studied before. To our knowledge, the current data are therefore the first evidence of a link between an increased protein/carbohydrate ratio and circulating 2-AG concentrations. Further research is needed to investigate the potential impact on weight loss and weight maintenance of this observation.

While AEA has been described as an orexigenic compound in the literature [20,36], PEA, OEA, and PREG were associated with anorexigenic effects [22,46,47,48,49]. OEA has been suggested to influence fat catabolism [50] and stimulate lipolysis [51]. While OEA was not associated with fat oxidation in the current study, the inverse relationship found between OEA and blunted TAG concentrations may be due to an increase in lipolysis.

In addition, in the present study, research participants were assessed in a controlled respiration chamber setting, and were fed in energy balance, which could overshadow the perception of hunger and satiety that people experience in a real-life situation. Finally, the fact that 2-AG and AEA behave differently should not be surprising, as they are synthetized through different pathways, which may further support a different role for the compounds in food intake regulation [52]. As for the origin of endocannabinoids and their related compounds, we and others have proposed that prandial-related changes in plasma AEA, 2-AG, OEA and PEA may primarily reflect changes in synthesis from the gastrointestinal tract [36].

Regarding the gut peptides, we found an inverse association between hunger and PYY concentrations in the HP/MCHO group only. PYY has previously been shown to be increased in response to a HP diet and was suggested to mediate the satiating effects of dietary protein [24]. Our data supports other literature proposing a ‘threshold’ level of protein to be necessary to exert effects on PYY-mediated impact on hunger [53,54]. PYY and GLP-1 concentrations have been linked to satiety and food intake [26], with rather weak associations [13]. GLP-1 release on the other hand has been primarily associated with dietary carbohydrates and fat and has been shown to be lower by high protein whilst satiety was increased compared to high carbohydrate [55]. Some evidence points to a stimulation of GLP-1 by a HP condition [56,57]. In comparison, PYY appeared to be stimulated by dietary protein content to a larger extent than GLP-1 [27,57]. However, changes in gut peptides by dietary protein content in the present study did not translate into changes in appetite perception. Relations between endogenous gut peptides and appetite ratings have shown hardly any, or conflicting results in the past [27], and have often been either absent [11], or weak [13]. Despite these discrepancies in subjective and objective appetite measures, appetite ratings by visual analogue scales have been shown to be highly reproducible [34] and may therefore still be important in assessing appetite next to physiologic measures. Taken together, the current study suggests that observed effects on 2-AG, hunger, and the association of PYY and hunger are nutrient-related, since they are especially shown in the HP/MCHO group. 

While changes in TAG concentrations were blunted in the high-protein/moderate-carbohydrate group, both intervention groups increased their TAG concentrations when comparing pre- and post-respiration chamber measurements. The general increase in TAG may be related to the sedentary circumstances the participants experienced throughout their 48 h stay [58,59]. High protein intake has previously been suggested to increase lipolysis [60] and inhibit lipogenesis [61], which may explain the blunted increase in TAG in the HP/MCHO condition compared to the MP/HCHO condition in the current study. 

The current study investigates the effects of a diet with higher protein and lower carbohydrate content compared to a diet with lower protein and higher carbohydrate content. Although there is some evidence describing the role of protein over carbohydrate on hunger perception [11], the current study design is not sufficient to clearly allocate the effect to one of the two macronutrients. To assign the described effects on 2-AG, hunger and PYY, and TAG, conclusively to a high protein or moderate carbohydrate level, or to a combination of both, longer follow-up studies are needed.

## 5. Conclusions

In conclusion, findings from the present study confirm that a higher dietary protein/carbohydrate ratio has appetite regulating effects. Additionally, it shows that 2-AG concentrations appear to be an important contributor to appetite related effects of a high- protein/moderate-carbohydrate diet, and that PYY may be one of the mediators in this increased protein/carbohydrate ratio-induced appetite regulation with an impact on reduced hunger perception, after 34 months in the post-obese phase. 

## Figures and Tables

**Table 1:** Subject characteristics of the moderate/high carbohydrate (MP/HCHO), and high-protein/moderate-carbohydrate (HP/MCHO) group at baseline of the respiration chamber experiment.

	MP/ HCHO (*n* = 18)	HP/MCHO (*n* = 20)
*N* (f/m)	18 (9/9)	20 (13/7)
Age (year)	65.1 ± 5.8	64.0 ± 6.2
BMI (kg/m^2^)	29.0 ± 3.8	28.9 ± 4.0
Body-fat (%)	39.5 ± 8.1	40.7 ± 7.7
Fat mass (kg)	33.9 ± 7.1	34.8 ± 8.8
Fat-free mass (kg)	52.5 ± 10.9	50.8 ± 11.3
Glucose (mmol/L)	5.8 ± 0.4	5.7 ± 0.5
Insulin (μU/mL)	14.8 ± 7.6	14.4 ± 4.9
HOMA-IR	3.8 ± 1.8	3.6 ± 1.3
TAG (mmol/L)	1.5 ± 0.7	1.2 ± 0.6 *

Values are means ± standard deviation (SD). BMI: Body Mass Index; HOMA-IR: Homeostatic Model Assessment of Insulin Resistance; TAG: triacylglycerol. * *p* < 0.05.

## Supplementary Materials

The following are available online at https://www.mdpi.com/2072-6643/11/10/2269/s1, Figure S1: Flow diagram of the study.

## Abbreviations

2-AG	2-arachidonoylglycerol
AEA	anandamide
AUC	area under the curve
BHB	β-hydroxybutyrate
BMI	body mass index
BMR	basal metabolic rate
CB1	cannabinoid receptor 1
CB2	cannabinoid receptor 2
dAUC	decremental area under the curve
EDTA	ethylenediaminetetraacetic acid
EI	energy intake
FFM	fat-free mass
FM	fat mass
GC-MS	gas chromatography-mass spectrometry
GC-MS/MS	gas chromatography-tandem mass spectrometry
GI	glycemic index
GLP-1	glucagon-like peptide-1
HOMA-IR	homeostatic model assessment for insulin resistance
HP/MCHO	high protein/ moderate carbohydrate
iAUC	incremental area under the curve
MJ	megajoule
mmVAS	milimeter visiual analogue scale
MP/ HCHO	moderate-protein/ high-carbohydrate
OEA	oleoylethanolamide
OGTT	oral glucose tolerance test
PEA	palmitoylethanolamide
PMSF	phenylmethylsulfonyl fluoride
PREG	pregnenolone
PYY	polypeptide YY
TAG	triacylglycerol
T2D	type II diabetes
VAS	visual analogue scale
