# Supplementary figures and images for: Effects of a High-Protein/Moderate-Carbohydrate Diet on Appetite, Gut Peptides, and Endocannabinoids—A Preview Study
Source: Nutrients. 2019 Sep 21;11(10):2269. doi: 10.3390/nu11102269 (PMC6835833; doi:10.3390/nu11102269)

**CONSORT 2010 Flow Diagram**

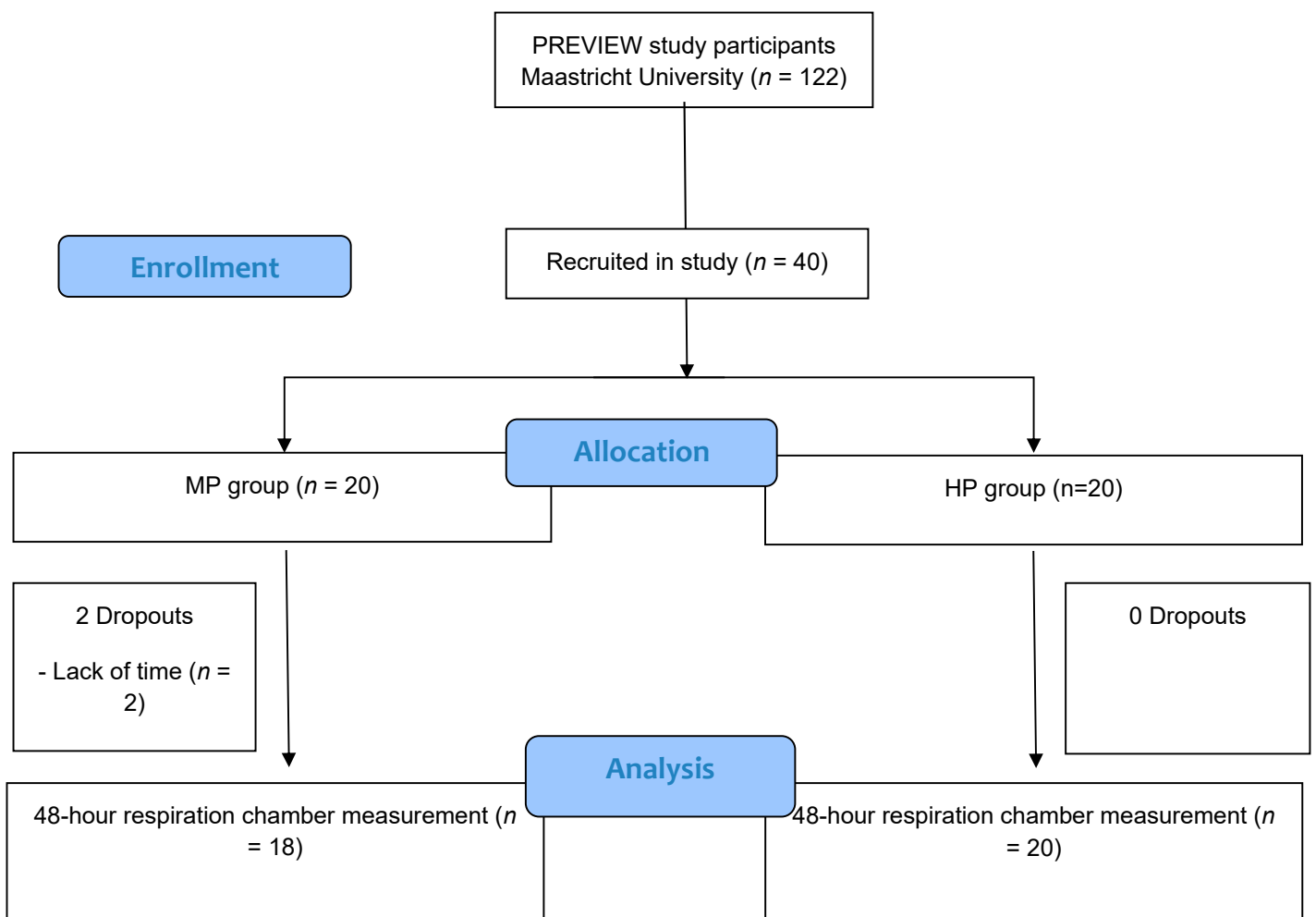

Figure S1: Flow diagram of study.

Supplement: Supplementary file 1 [file nutrients-11-02269-s001.pdf]
